# ^1^H, ^13^C, and ^15^N backbone chemical shift assignments of the nucleic acid-binding domain of SARS-CoV-2 non-structural protein 3e

**DOI:** 10.1007/s12104-020-09971-6

**Published:** 2020-08-08

**Authors:** Sophie M. Korn, Karthikeyan Dhamotharan, Boris Fürtig, Martin Hengesbach, Frank Löhr, Nusrat S. Qureshi, Christian Richter, Krishna Saxena, Harald Schwalbe, Jan-Niklas Tants, Julia E. Weigand, Jens Wöhnert, Andreas Schlundt

**Affiliations:** 1 Institute for Molecular Biosciences, Johann Wolfgang Goethe-University Frankfurt, Max-von-Laue-Str. 9, 60438 Frankfurt, Germany; 2 Institute for Organic Chemistry and Chemical Biology, Johann Wolfgang Goethe-University Frankfurt, Max-von-Laue-Str. 7, 60438 Frankfurt, Germany; 3 Institute of Biophysical Chemistry, Johann Wolfgang Goethe-University Frankfurt, Max-von-Laue-Str. 9, 60438 Frankfurt, Germany; 4 Center for Biomolecular Magnetic Resonance (BMRZ), Johann Wolfgang Goethe-University Frankfurt, 60438 Frankfurt, Germany; 5 Department of Biology, Technical University of Darmstadt, Schnittspahnstr. 10, 64287 Darmstadt, Germany

**Keywords:** SARS-CoV-2, Non-structural protein, Nucleic acid-binding domain, Solution NMR-spectroscopy, Protein drugability, Covid19-NMR

## Abstract

The ongoing pandemic caused by the *Betacoronavirus* SARS-CoV-2 (Severe Acute Respiratory Syndrome Coronavirus-2) demonstrates the urgent need of coordinated and rapid research towards inhibitors of the COVID-19 lung disease. The *covid19-nmr* consortium seeks to support drug development by providing publicly accessible NMR data on the viral RNA elements and proteins. The SARS-CoV-2 genome encodes for approximately 30 proteins, among them are the 16 so-called non-structural proteins (Nsps) of the replication/transcription complex. The 217-kDa large Nsp3 spans one polypeptide chain, but comprises multiple independent, yet functionally related domains including the viral papain-like protease. The Nsp3e sub-moiety contains a putative nucleic acid-binding domain (NAB) with so far unknown function and consensus target sequences, which are conceived to be both viral and host RNAs and DNAs, as well as protein-protein interactions. Its NMR-suitable size renders it an attractive object to study, both for understanding the SARS-CoV-2 architecture and drugability besides the classical virus’ proteases. We here report the near-complete NMR backbone chemical shifts of the putative Nsp3e NAB that reveal the secondary structure and compactness of the domain, and provide a basis for NMR-based investigations towards understanding and interfering with RNA- and small-molecule-binding by Nsp3e.

## Biological context

SARS-CoV-2, the cause of the early 2020 pandemic accompanied by the respiratory disease called COVID-19, is the latest representative of the *coronaviridae* family, which also comprises the 2002 first generation SARS-CoV and the Middle East Respiratory Syndrome (MERS)-CoV. The severe velocity of virus spread, based on its unexpectedly high infectivity, demands for a rapid action towards both the development of a vaccine and potent viral inhibitors to weaken or eliminate symptoms that are a major life-thread, especially to older generations worldwide.

The almost 30-kb enveloped positive-sense single-stranded RNA of SARS-CoV-2 represents one of the largest known viral genomes. Contained therein are possible 14 open reading frames (ORFs) that encode for up to 30 transcripts, the majority of which have been proven at protein level (Gordon et al. 2020). Within the highly conserved proteins of Betacoronaviruses (Yoshimoto 2020), the ORF1a/b-encoded non-structural proteins (Nsp) 1–16 assemble the replication/transcription complex which comprises an incompletely understood network of viral-viral and host-viral protein-protein and RNA-protein interactions. Besides the structural Spike protein, important for viral entry, it is a set of non-structural proteins that represent the canonical protein drug targets, among them the two proteases Nsp5 (Mpro) and Nsp3d (PLpro), the Nsp3b ADP-ribose-phosphatase macrodomain, and the Nsp7/8/12 RNA-dependent RNA polymerase complex.

Nsp3, the largest Nsp (Snijder et al. 2003), is one of the most enigmatic coronavirus proteins as it is composed of a plethora of functionally related, yet independent subunits. After cleavage of Nsp3 from the full-length ORF1-encoded polypeptide chain, it displays a 1945-residue multi-domain protein, with individual functional entities that are sub-classified from Nsp3a to Nsp3e followed by the ectodomain embedded in two transmembrane regions and the very C-terminal CoV-Y domain. Nsp3e is unique to Betacoronaviruses and consists of a nucleic acid-binding domain (NAB) and the so-called *group 2-specific marker* (G2M) (Neuman et al. 2008). Structural information is rare; while the G2M is predicted to be intrinsically disordered (Lei et al. 2018), the only available experimental structure of the Nsp3e NAB was solved from SARS-CoV by the Wüthrich lab using solution NMR (Serrano et al. 2009). The SARS-CoV Nsp3e NAB was shown to bind G-rich ssRNA and to possess DNA-unwinding capability (Neuman et al. 2008), while its precise function and well-defined consensus target sequences have remained unknown. Seeing its specific appearance, Nsp3e thus represents a potential drug target for both the current as well as potential future Betacoronavirus epidemic waves.

The 2020 founded research consortium *covid19-nmr* seeks to rapidly and publicly support the search for anti-viral drugs using an NMR-based screening approach which requires the broad production of all drugable proteins and RNAs and their as comprehensive as possible assignment of NMR resonances, and eventually the determination of structures to be used in rational drug design. We here provide the near-complete backbone assignment of the SARS-CoV-2 Nsp3e NAB and thereby enable its exploitability in follow-up applications, such as residue-resolved drug screening and interaction mapping.

## Methods and experiments

### Construct design

This study uses the SARS-CoV-2 NCBI reference genome entry NC_045512.2, identical to GenBank entry MN908947.3 (Wu et al. 2020). The definition of domain boundaries for the Nsp3e NAB was guided by the available NMR structure (PDB 2K87) of its closest homologue, i.e. Nsp3e from the 2002 first generation SARS-CoV (Serrano et al. 2009), sharing 82% sequence identity. Based on the sequence alignment of the entire SARS-CoV-2 Nsp3e with SARS-CoV Nsp3e and consideration of flexible overhangs observed in the structure, we defined our expression construct to span amino acids 1088–1203 counting the overall Nsp3 primary sequence. A codon-optimized expression construct of SARS-CoV-2 Nsp3e NAB was obtained from GenScript Biotech (Netherlands), inserted into the pET3b-based vector pKM263, containing an N-terminal His_6_-tag, a GST-tag and a tobacco etch virus (TEV) cleavage site. Due to the nature of the TEV cleavage site, the produced protein contained four artificial N-terminal residues (Gly-3, Ala-2, Met-1 and Gly0) after cleavage, before the original protein sequence starts with Tyr1 according to Tyr1088 in the full-length Nsp3 sequence.

### Sample preparation

Uniformly ^13^C,^15^N-labelled Nsp3e NAB protein was expressed in *E*. *coli* strain BL21 (DE3) in M9 minimal medium containing 1 g/L ^15^NH_4_Cl (Cambridge Isotope Laboratories), 2 g/L ^13^C_6_-d-glucose (Eurisotop) and 100 µg/mL ampicillin. Protein expression was induced at O.D. 600_nm_ of 0.7 with 1 mM isopropyl-beta-thiogalactopyranoside for 18 h at room temperature. The cell pellet was resuspended in 50 mM sodium phosphate, 300 mM sodium chloride, 10 mM imidazole, 2 mM Tris-(2-carboxyethyl)-phosphine (TCEP) and 100 µL protease inhibitor mix (SERVA) per 1 L of culture, pH 6.5. Cells were disrupted by sonication. The supernatant was cleared by centrifugation (20 min, 7000×*g*, 4 °C). The cleared supernatant was passed over a Ni^2+^-NTA gravity flow column (Sigma-Aldrich) and the His_6_-GST-tag was cleaved over night at 4 °C with 0.5 mg of TEV protease per 1 L of culture, while dialyzing into size exclusion buffer (25 mM sodium phosphate, 150 mM sodium chloride, 2 mM DTT, 0.02% NaN_3_, pH 7.0). TEV protease and the cleaved tag were removed via a second Ni^2+^-NTA gravity flow column and Nsp3e was further purified via size exclusion on a HiLoad 16/600 SD 75 (GE Healthcare). Fractions containing pure Nsp3e were determined by SDS-PAGE, pooled and concentrated using Amicon centrifugal concentrators (molecular weight cutoff 3 kDa). NMR samples were prepared in 25 mM sodium phosphate pH 7.0, 150 mM sodium chloride, 2 mM TCEP, 0.02% NaN_3_, 10% (v/v) D_2_O, 300 µM 4,4-dimethyl-4-silapentane-1-sulfonic acid (DSS) as internal chemical shift standard at Nsp3e concentrations of 0.6 to 1.1 mM.

### NMR experiments

The Nsp3e NAB backbone assignment was performed by analyzing [^15^N,^1^H]-HSQC and [^15^N,^1^H]-TROSY experiments, the triple-resonance experiments HNCACB and HN(CO)CACB, and verified by the HN(CA)CO/HNCO pair of spectra. Semi-constant-time (^15^N) triple-resonance pulse sequences applied in this study were [^15^N,^1^H]-TROSY-based (Pervushin et al. 1997; Salzmann et al. 1998) and used sensitivity-enhanced gradient echo/antiecho coherence selection (Czisch and Boelens 1998; Schulte-Herbrüggen and Sorensen 2000). Acceleration of longitudinal ^1^H relaxation between scans was achieved in the Band-Selective Excitation Short-Transient (BEST) (Lescop et al. 2007; Schanda et al. 2006; Solyom et al. 2013) manner using exclusively shaped proton pulses with bandwidths/offsets of 4.8/8.3 ppm and the inter-scan delay set to 0.3 or 0.4 s. A 3D NOESY-[^15^N,^1^H]-HSQC (Marion et al. 1989; Zuiderweg and Fesik 1989) with water suppression using a WATERGATE sequence (Piotto et al. 1992) was recorded to complete backbone assignments and also provided Gln/Asn NH_2_ group assignments.

Sequence-specific assignments of tryptophan side chain ^1^H^ε1^/^15^N^ε1^ resonances were obtained with a [^15^N,^1^H]-BEST-TROSY version of the HN(CDCG)CB experiment (Lohr and Ruterjans 2002) with proton pulses centered at 10 ppm and covering a bandwidth of 4 ppm. A slowly exchanging histidine imidazole ^1^H^Nε2^ resonance was assigned using a 2D BEST-TROSY-H(NCDCG)CB version with the magnetization transfer pathway adapted to histidine side chains and proton pulses centered at 12 ppm (Andersson et al. 1998). The ^15^N heteronuclear NOE experiment was performed as an interleaved pseudo-3D TROSY version (Lakomek et al. 2012) using 256 indirect complex points. All NMR experiments were carried out at a sample temperature of 25 °C using Bruker Avance spectrometers of 600, 700 and 950 MHz proton Larmor frequency, equipped with cryogenic z-axis gradient probes. Data acquisition and processing was undertaken using Topspin versions 3 and 4. Cosine-squared window functions were applied for apodization in all dimensions. Spectra were referenced with respect to internal DSS and for ^13^C and ^15^N as described in (Wishart et al. 1995).

### Assignments and data deposition

All assignments of the Nsp3e NAB were performed using the CCPNMR analysis 2.4 software suite (Vranken et al. 2005) and the program Sparky (Lee et al. 2015).

**Fig. 1:**
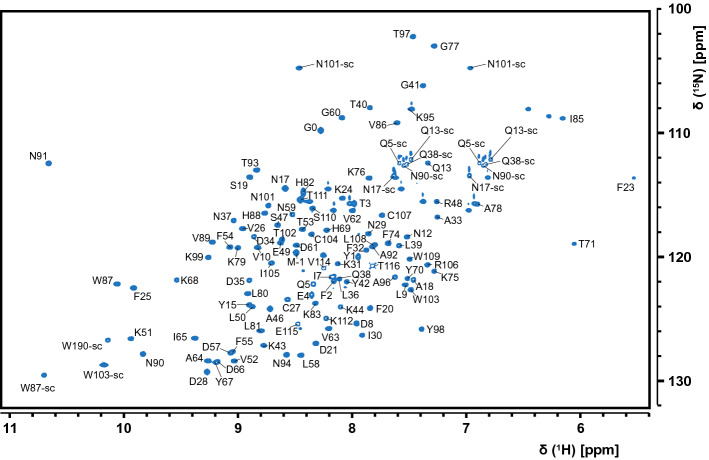
^1^H, ^15^N-HSQC spectrum of the ^13^C, ^15^N-labelled SARS-CoV-2 Nsp3e NAB at 1.1 mM concentration in 25 mM sodium phosphate pH 7, 150 mM NaCl, 2 mM TCEP, 0.02% NaN_3_, and 0.3 mM DSS collected at 298 K on a 600 MHz Bruker Avance II spectrometer equipped with a triple-resonance TCI cryogenic probe. Backbone NH peaks are labelled with their assignments. Trp, Gln and Asn sidechain amides are indicated by W-sc, Q-sc and N-sc, respectively

The Nsp3e NAB ^1^H,^15^N-HSQC shown in Fig. 1 shows an excellent peak dispersion. Of note, we obtained a yet better resolved amide correlation spectrum at 950 MHz proton frequency; however, we found some resonances exchange broadened and only visible at lower field strength, e.g. Phe23. For convenience, residues were numbered starting with 1 on Tyr1088. The overall high quality of all spectra allowed the assignment of > 98% of all backbone amides within the natural sequence (Tyr1-Thr116, according to Tyr1088-Thr1203), all Trp and Gln sidechain amides, and 3 out of 10 Asn sidechain amides (17, 90, 101). The assignments are in good agreement with the previously published assignments of the 2002 SARS-CoV Nsp3e NAB_1066 − 1181_ (Serrano et al. 2008), which reflects the high sequence similarity (Yoshimoto 2020). Only two residues of the natural sequence (Asn22 and Ser73, both likely in flexible loop regions) could not be assigned in their backbone amides due to obvious line-broadening beyond detectability, which notably had also been observed for the SARS-CoV Nsp3e (Serrano et al. 2008). For amino acids Glu5, Ile7, Asn8, Asp57, Leu58, and Val114 we observed a second, minor conformation based on the preceding prolines with both *cis* and *trans* isomers present.

**Fig. 2:**
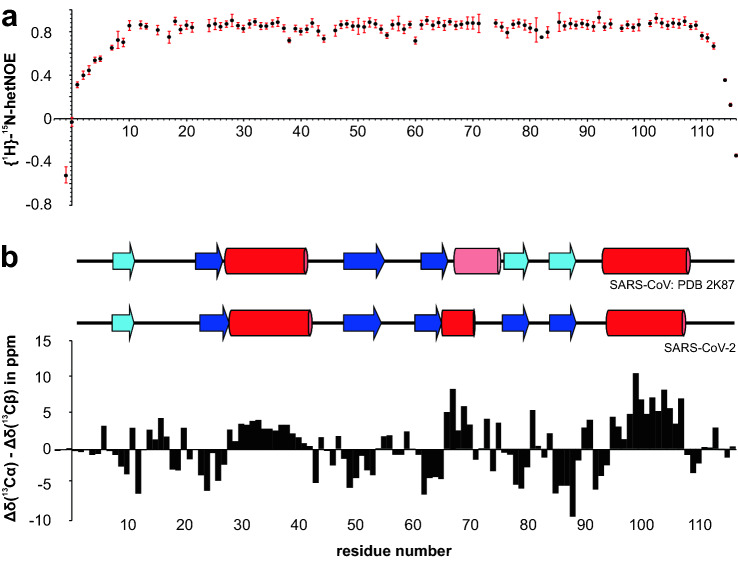
Display of {{^1^H}}^15^N heteronuclear NOE values (**a**) and combined Cα/Cβ carbon secondary chemical shift (SCS) values of the SARS-CoV-2 Nsp3e NAB plotted against the protein primary sequence (**b**). **a** hetNOE values are shown with errors as derived from the program CCPNMR Analysis 2.4 (Vranken et al. 2005) based on the respective signal-to-noise of spectra. No values are shown for Asn22 and Ser73 (missing assignments) and Phe23, His82 and Lys95 due to large relative errors based on the overall low peak intensities of these amides. Additional gaps derive from prolines. **b** SCS are interpreted towards their underlying secondary structure as shown above the panel (experimental) and when compared to the SARS-CoV Nsp3e homologue structure (Serrano et al. 2008, 2009) from PDB entry 2K87. α-helices are shown with red bars, β-strands with blue arrows, respectively. Light colors indicate the presence of elements with imperfect geometry in the structure or merely tentative secondary chemical shifts

To assess the overall compactness of the NAB and internal dynamics, we recorded hetNOE data (Fig. 2a) as a function of the primary sequence. For residues 8-109, hetNOE values of 0.65 or higher were measured indicating an overall rigid structure of the protein. No regions of increased flexibility were observed except for the two termini (residues 1–7 and 110–116). We also calculated carbon secondary chemical shifts based on the chemical shifts of C_α_ and C_β_ (Fig. 2b bottom) relative to random coil values essentially as described by (Wishart and Sykes 1994). Four consecutive residues with significant negative or positive shifts were used to define either β-strands or α-helices, respectively. Our data suggest a ββαββαββα-fold, which is in agreement with the structure of its homologue from SARS-CoV (Fig. 2b top). While all secondary structure elements well align between the two homologues, helix-2 - according to our data - is shorter and directly connects to β-strand 4. The very terminal residues do not display secondary structure content, which is in line with the increased flexibility observed in the hetNOE experiment. Our data thus suggest that the NAB of SARS-CoV-2 Nsp3e resembles a similar structure as the SARS-CoV Nsp3e (Serrano et al. 2009). Our determined NMR resonance assignments and spectral quality clearly prove the Nsp3e NAB drugability and will now pave the way towards a solution structure, RNA- and protein interaction studies, and residue-resolved high-throughput drug screening as a crucial contribution to the initiative of screening all potential SARS-CoV-2 proteins.

## Data Availability

The chemical shift values for the ^1^H, ^13^C and ^15^N resonances of SARS-CoV-2 Nsp3e have been deposited at the BioMagResBank (https://www.bmrb.wisc.edu) under accession number 50334. Spectral raw data (upon request) and assignments are also accessible through https://covid19-nmr.de.
